# Endometrial Stromal Nodule: A Rarity and a Pathological Challenge

**DOI:** 10.1155/2015/376817

**Published:** 2015-06-28

**Authors:** Camilla Skovvang Borg, Peter Humaidan, Hanne Noer, Huda Galib Majeed

**Affiliations:** ^1^Department of Obstetrics and Gynecology, Viborg Regional Hospital, 8800 Viborg, Denmark; ^2^The Fertility Clinic, Skive Regional Hospital, 7800 Skive, Denmark; ^3^Faculty of Health, University of Aarhus, 8000 Aarhus, Denmark; ^4^Department of Pathology, Viborg Regional Hospital, 8800 Viborg, Denmark

## Abstract

Endometrial stromal tumors are rare, and endometrial stromal nodule is the least common. In the region of Middle Jutland, Denmark, only two cases are reported since 1995. The nodules are benign; nevertheless, hysterectomy is the treatment of choice. Tumor margins are required for diagnosis and essential to differentiate it from an invasive stromal sarcoma whose prognosis is very different. We report a rare case of a 38-year-old woman, with presurgical diagnosis of a uterine tumor/polyp. She presented with nausea and changes in bleeding pattern and initially had a transcervical polyp resection performed. Histopathological examination showed the presence of an endometrial stromal tumor with unclear margins, and an invasive malignant endometrial sarcoma could not be excluded. Pathological examination revealed an endometrial stromal nodule with invasion, not exceeding three mm. Endometrial stromal tumors are interesting due to their rare existence and difficulties in establishing a histological diagnosis. Although endometrial stromal nodules are benign entities, they must be differentiated from the other invasive malignant stromal tumors, which may change the final prognosis. No preoperative diagnostic tools are at hand, and benign as well as malignant tumors are treated with hysterectomy.

## 1. Introduction

Endometrial stromal tumors (ESTs) are rare. The 2014 WHO classification scheme incorporates recent molecular findings into the classification, dividing ESTs into endometrial stromal nodule (ESN), low-grade endometrial stromal sarcoma (LGESS), high-grade endometrial stromal sarcoma (HGESS), and undifferentiated uterine sarcoma (UUS) [1] based on their histological appearance. However, the differentiation between the subtypes is difficult [2, 3] in specimens obtained after curettage. Furthermore, the prognosis varies from benign to invasive and malignant tumors. Thus, a histological examination of the uterus is the most accurate method of diagnosis and the recommended therapy of an endometrial stromal neoplasm is a total hysterectomy.

The overall incidence of ESS in Scandinavia is 0.3 per 100,000 [4].

*ESN* is the least common type of endometrial stromal tumors [5] characterized and defined as benign and noninvasive. Since 1995, only two cases have been reported in Central Jutland, Denmark, in a population of 1.2 million.

Tavassoli and Norris and Dionigi et al., being the two largest published series, showed no recurrences after a follow-up period of up to 16 years and 17.8 years, respectively [6, 7]. The age range for ESNs is wide, from 31 years to 86 years with a mean of 54 years [6], and the patients usually present with abnormal vaginal bleeding.

Given the rarity of these tumors, there are limited reports in the literature concerning the clinical management and final outcome of these cases. Some report experimental fertility preserving treatment and others discuss the pathological difficulties, distinguishing benign types from malignant types.

## 2. Case

We here report a rare case of ESN. A 38-year-old woman was referred to the department with nausea and abnormal uterine bleeding. She was a 2 para 2 gravida and wanted to obtain an additional pregnancy. A transvaginal ultrasound scan (TVUS) revealed a suspected intrauterine polyp and fibroma measuring 11 × 45 mm. A transcervical resection of the polyp was performed (not polypectomy). The histopathological examination showed the presence of an endometrial stromal tumor with unclear margins, and an invasive malignant endometrial sarcoma could not be excluded. The preoperative MRI showed an invasive tumor in the endometrium (Figures 3(a) and 3(b)), however, without signs of extra uterine spreading. A fertility preservation treatment was discussed with the patient, but the patient was advised to go through with hysterectomy. Subsequently, she underwent a successful total laparoscopic hysterectomy.

## 3. Pathology

In general, endometrial stromal tumors are very rare, accounting for 3% of all uterine neoplasms [8]. These tumors are characterized primarily by the tumor invasiveness and degree of stromal differentiation. However, both ESS and ESN can appear histologically similar with distinction made only after evaluation of the full hysterectomy specimen; findings of myometrial or vascular invasion less than 3 mm readily make the diagnosis of ESN. ESNs show focal smooth muscle differentiation and express CD10 and hormone receptors 8.

**Figure 1:**
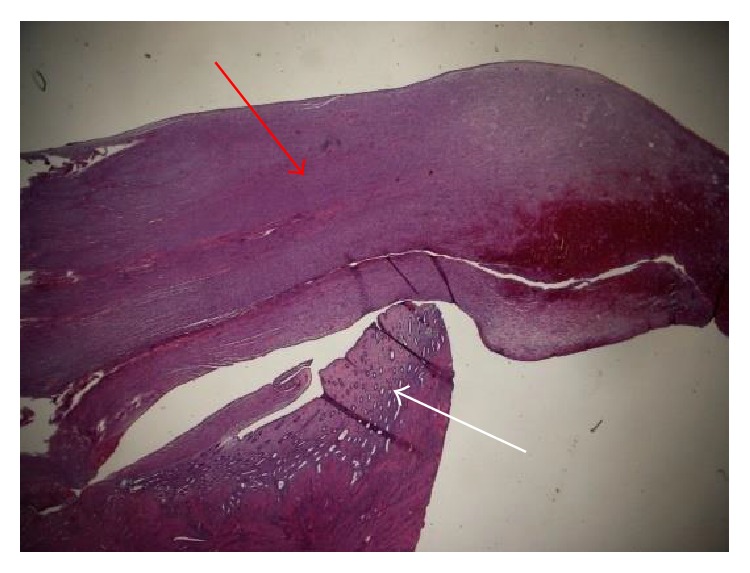
Macroscopic view. Note the sharp demarcation with normal myometrium (white arrow: normal myometrial tissue; red arrow: stromal nodular tissue).

**Figure 2:**
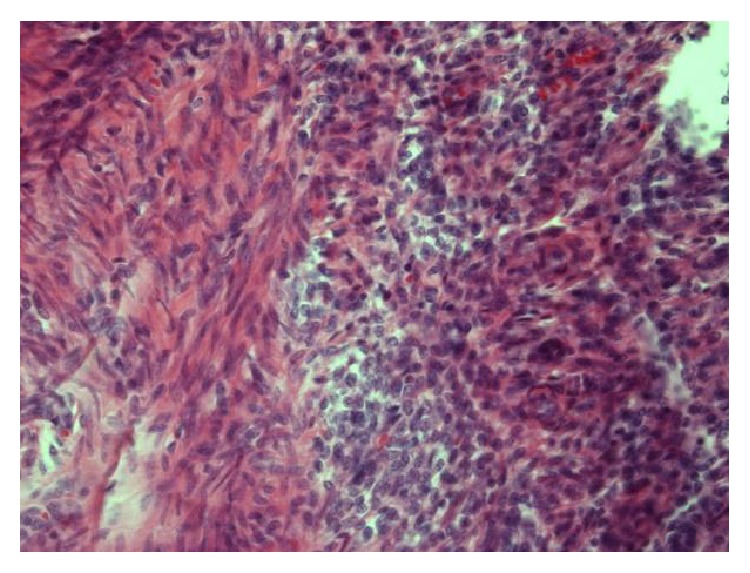
Endometrial stromal nodule with smooth muscle differentiation. Smooth muscle on the left and nodulus (compact tissue) on the right.

**Figure 3:**
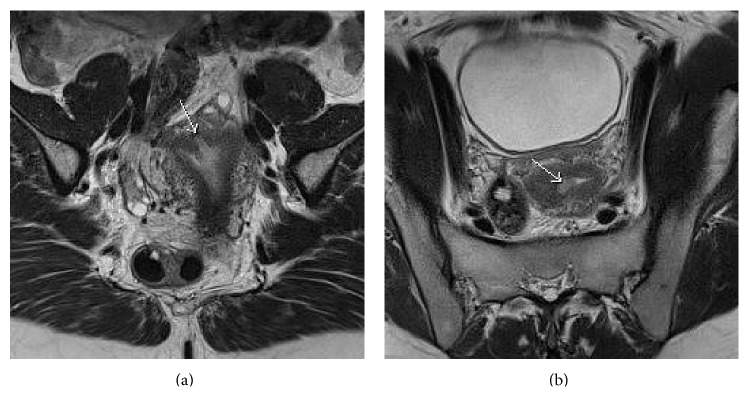
Coronal (a) and axial (b) T2WI MRI shows endometrial mass (white arrow) invading the myometrium.

Pathological examination of the removed uterus in the present case revealed an ESN measuring 19 mm with an invasion of two mm. Cellular atypia was present with slightly elevated mitotic count (six per ten high-power fields). The tumor was well defined against the underlying myometrium (Figures 1 and 2) in the part of the fundus, but, in other places, it was more irregular with finger-like projections into the myometrium, however not exceeding three mm. Immunohistochemical analysis showed high positivity for CD10 but showed in a lesser extent positivity for smooth muscle myosin.

Based on morphology and spreading, the tumor is classified as an endometrial stromal nodulus.

## 4. Discussion

Hysterectomy is the gold standard in cases with ESN and low-grade endometrial stromal tumors, considering their theoretic ability to infiltrate and become malignant [3]. Until now, no immunohistochemical biomarker has proven to be able to distinguish benign nodules from potential malignant sarcomas prior to hysterectomy.

A small study from 2005 showed a higher frequency of MIB-1 and a lower estrogen/progesterone receptor expression in endometrial stromal sarcomas than in endometrial stromal nodules [9], suggesting that these biomarkers will be able theoretically to distinguish between the types and thereby allow a more conservative treatment when diagnosed with a benign type.

Previously, Schilder et al. [10] published a case report with successful hormonal therapy of an endometrial stromal nodule, allowing conservative management in a young nulliparous woman, obviously with the aim to preserve reproductive function. A few case reports have shown promising results, using fertility preserving treatments such as local excision, endocrine therapy, and photodynamic therapy in young women with low-grade endometrial stromal sarcomas [11, 12]. However, the shortage of randomized controlled trials limits the clinical implementation. Further studies are clearly needed to draw conclusions.

In the present case, the patient was advised to have a total hysterectomy performed, although she still hoped for an additional pregnancy. This advice was given based on the current diagnostic available tools and the lack of knowledge regarding long-term consequences of a conservative observational treatment.

## 5. Conclusion

Although rare, endometrial stromal tumors are clinically important due to difficulties establishing the histological diagnosis. No preoperative imaging can completely rule out malignancy. Despite ESNs being benign entities, hysterectomy is still the gold standard as a total histological examination of the uterus is necessary to exclude malignancy and no immunohistochemical biomarkers have been proven yet to be useful. Our molecular understanding of these tumors is still in progress and the identification of genetic alterations [13] has increased our ability to distinguish the endometrial stromal neoplasms. Unfortunately, these have not yielded significant improvement in our treatment approach to these tumors. How to manage these rare tumors and how to ensure patients benefits of today's precision therapy requires more work, especially in young nulliparous women, for whom reproduction issues are still present. Unfortunately, the rarity of these tumors makes randomized controlled trials nearly impossible to perform. In our case, the patient had two children and the risk of a bad prognosis was higher than the wishes for an additional pregnancy.
